# Self-assembled fluorescent hybrid nanoparticles-mediated collaborative lncRNA CCAT1 silencing and curcumin delivery for synchronous colorectal cancer theranostics

**DOI:** 10.1186/s12951-021-00981-7

**Published:** 2021-08-11

**Authors:** Fan Jia, Yunhao Li, Xiongwei Deng, Xuan Wang, Xinyue Cui, Jianqing Lu, Zian Pan, Yan Wu

**Affiliations:** 1 CAS Key Laboratory for Biomedical Effects of Nanomaterials and Nanosafety, CAS Center for Excellence in Nanoscience, National Center for Nanoscience and Technology, Beijing, 100190 China; 2 Department of General Surgery, Peking Union Medical College Hospital, Peking Union Medical College, Chinese Academy of Medical Sciences, Beijing, 100730 People’s Republic of China; 3 University of Chinese Academy of Sciences, Beijing, 100049 People’s Republic of China

**Keywords:** LncRNA CCAT1, Curcumin, Combinational therapy, Co-delivery, RNAi, Theranostic nanoparticles

## Abstract

**Background:**

Cancer synergistic therapy strategy in combination with therapeutic gene and small molecule drug offers the possibility to amplify anticancer efficiency. Colon cancer-associated transcript-1 (CCAT1) is a well identified oncogenic long noncoding RNA (lncRNA) exerting tumorigenic effects in a variety of cancers including colorectal cancer (CRC).

**Results:**

In the present work, curcumin (Cur) and small interfering RNA targeting lncRNA CCAT1(siCCAT1) were co-incorporated into polymeric hybrid nanoparticles (CSNP), which was constructed by self-assembling method with two amphiphilic copolymers, polyethyleneimine-poly (d, l-lactide) (PEI-PDLLA) and 1,2-distearoyl-sn-glycero-3-phosphoethanolamine-*N*-[methoxy (polyethylene glycol) (DSPE-mPEG). Owing to the multicolor fluorescence characteristics of PEI-PDLLA, the constructed CSNP could be served as a theranostic nanomedicine for synchronous therapy and imaging both in vitro and in vivo. Resultantly, proliferation and migration of HT-29 cells were efficiently inhibited, and the highest apoptosis ratio was induced by CSNP with coordination patterns. Effective knockdown of lncRNA CCAT1 and concurrent regulation of relevant downstream genes could be observed. Furthermore, CSNP triggered conspicuous anti-tumor efficacy in the HT-29 subcutaneous xenografts model with good biosafety and biocompatibility during the treatment.

**Conclusion:**

On the whole, our studies demonstrated that the collaborative lncRNA CCAT1 silencing and Cur delivery based on CSNP might emerge as a preferable and promising strategy for synergetic anti-CRC therapy.

**Graphic abstract:**

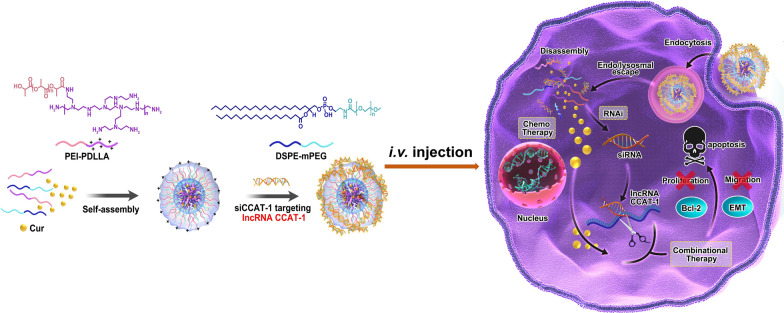

**Supplementary Information:**

The online version contains supplementary material available at 10.1186/s12951-021-00981-7.

## Introduction

Colorectal cancer (CRC) is one of the most commonly diagnosed cancer in both male and female [1]. The metastatic relapse is hard to be completely eliminated during surgery and usually resistant to chemotherapy, leading to high morbidity and mortality of CRC, although radical resection is the cornerstone of CRC treatment clinically [2, 3]. Nowadays, many types of epigenetic modifications have been found to be involved in the development of CRC. A new consensus on the evolutionary properties of CRC shifted the therapeutic paradigm to the development of new therapies for novel molecular targets related to the complex biological characteristics of the disease. Therefore, we believe that the use of gene therapy in combination with chemotherapy drugs provides broad prospects for CRC treatment.

Noncoding RNAs (ncRNAs) used to be considered as transcription junk since they lack of significant protein-coding capacity. However, more and more studies have shown that ncRNAs closely related to human diseases and widely involved in important biological processes in life activities, such as the development, and reproduction of individuals, apoptosis, and reprogramming of cells, etc. [4–7]. At present, long noncoding RNAs (lncRNAs) are operationally defined as a new class of ncRNAs that genes larger than 200 base-pairs, and many of which are uniquely expressed in differentiated tissues or specific cancer types [8, 9]. Colon cancer-associated transcript-1 (CCAT1), a recently identified oncogenic lncRNA, which maps to chromosome 8q24.21 containing single-nucleotide polymorphisms with a length of 2628 nucleotides, has been reported to be continuously raised in a variety of cancer tissues, especially in CRC [10–13]. CCAT1 has been confirmed that it participated in multiple processes related to the occurrence of tumor through aberrant expressions such as cell proliferation, apoptosis, migration, and invasion [14–18]. Zhang et al*.* indicated that silencing of CCAT1 brought out the regulation of G0/G1 arrest markers, Caspase-3 and B-cell lymphoma-2 (Bcl-2), p16, p21 and p27, and pro-apoptotic factors, which showed that knockdown of CCAT1 could suppress cancer cell proliferation by the motivation of G0/G1 arrest and apoptosis [19]. Luo et al*.* reported that the effect of CCAT1 on cancer cell migration and invasion might be regulated through the variation of epithelial-mesenchymal transition (EMT) because knockdown of CCAT1 would change the content of a collection of EMT-related genes [20]. Comparing to protein-coding genes, lncRNAs do not encode the protein, and thus their effect would emerge shortly after delivery and might lead to fewer side-effects [21–23]. For this reason, lncRNAs can be operated by RNA interference (RNAi) or other means with ease. RNAi-mediated gene silencing specificity is a prospective strategy to treat various diseases, including cancer.

Curcumin (Cur), is a natural extraction of turmeric, which has been confirmed to have the anti-tumor capacity, including the resistance of development, metastasis, and progression in multiple cancer types, such as CRC, pancreatic cancer, myeloid leukemia, and breast cancer [24–27]. Cur brings its anti-cancer effects into play by targeting multiple intracellular signaling pathways. Cur can inhibit EMT in breast cancer cells by blocking related genes indicated by in vitro examination, and the same suppression effect has been identified in pancreatic cancer cells [28, 29]. It has been confirmed that Cur induces apoptosis of various cancer cells including CRC cancer cells in response to the expression of Bcl-2 and Caspase-3, which is related to the sensitivity of CRC cancer cells to the chemotherapy [30, 31]. There is growing evidence that Cur has a multiplex scope of molecular targets, which supports the notion that Cur can trigger a cascade of many molecular interactions and biochemical reactions.

Over the years, combinational therapy has been applied in clinics that have addressed the problems related to single-drug treatment of tumors. Combinational therapy generally refers to the simultaneous co-delivery of multiple therapeutic agents or combining a different kind of therapies, such as chemotherapy, gene therapy, photothermal therapy, immunotherapy, and radiotherapy [32–36]. In recent years, the combinational therapy of chemotherapeutics and genes, whose targets are the same kind of cells, co-delivery with nanoparticles has become a new research focus. Different biological signal transduction pathways can be counteracted synergistically through combinations of two or more bioactive agents, to achieve a low dosage of each agent or confer context-specific multi-target mechanisms to overcome side-effects associated with resistance and high doses of a single medication [37–40]. In addition, the overall therapeutic advantage of the combination bioactive agents was found to be greater than the sum of the efficacy of the individual agents, because the co-delivered bioactive agents, which can target the same cellular signal pathways, could performance synergistically for higher therapeutic efficacy and better targeting selectivity [41, 42].

In this study, we constructed Cur and small interfering RNA CCAT1(siCCAT1) co-delivered nanoparticles (CSNP), which self-assembled from 1,2-distearoyl-sn-glycero-3-phosphoethanolamine-*N*-[methoxy (polyethylene glycol)] (DSPE-mPEG) and the synthesized amphiphilic copolymer polyethyleneimine-poly (d, l-lactide) (PEI-PDLLA) by film dispersion and electrostatic interaction methods, to achieve RNAi-chemo modulation combinational therapy against CRC. The in vitro experimental data implied that CSNP could encapsulate and delivered Cur and siCCAT1 to HT-29 cells effectively, which led to the down-regulation of CCAT1 and the influence of upstream and downstream genes related to CCAT1. Based on this, the inhibition of proliferation and migration, and the enhancement of apoptosis on HT-29 cells were observed. Furthermore, CSNP could be used as imaging agents at the cellular and living levels. Based on these results of in vivo fluorescence/photoacoustic imaging, the fluorescence/photoacoustic signals preferentially occurred in the tumor site, which suggested that CSNP could mainly accumulate at the tumor area after intravenous injection. In addition, due to the combinational therapy of Cur and siCCAT1, which had good biocompatibility in HT-29 xenograft mouse tumor models, CSNP demonstrated extraordinary anti-tumor efficacy. Taking all these factors into consideration, the results obtained indicate that CSNP could be used as an effective strategy of combined RNAi-chemo therapy for CRC.

## Results and discussion

### Construction and characterization of the co-delivery system CSNP

**Scheme 1:**
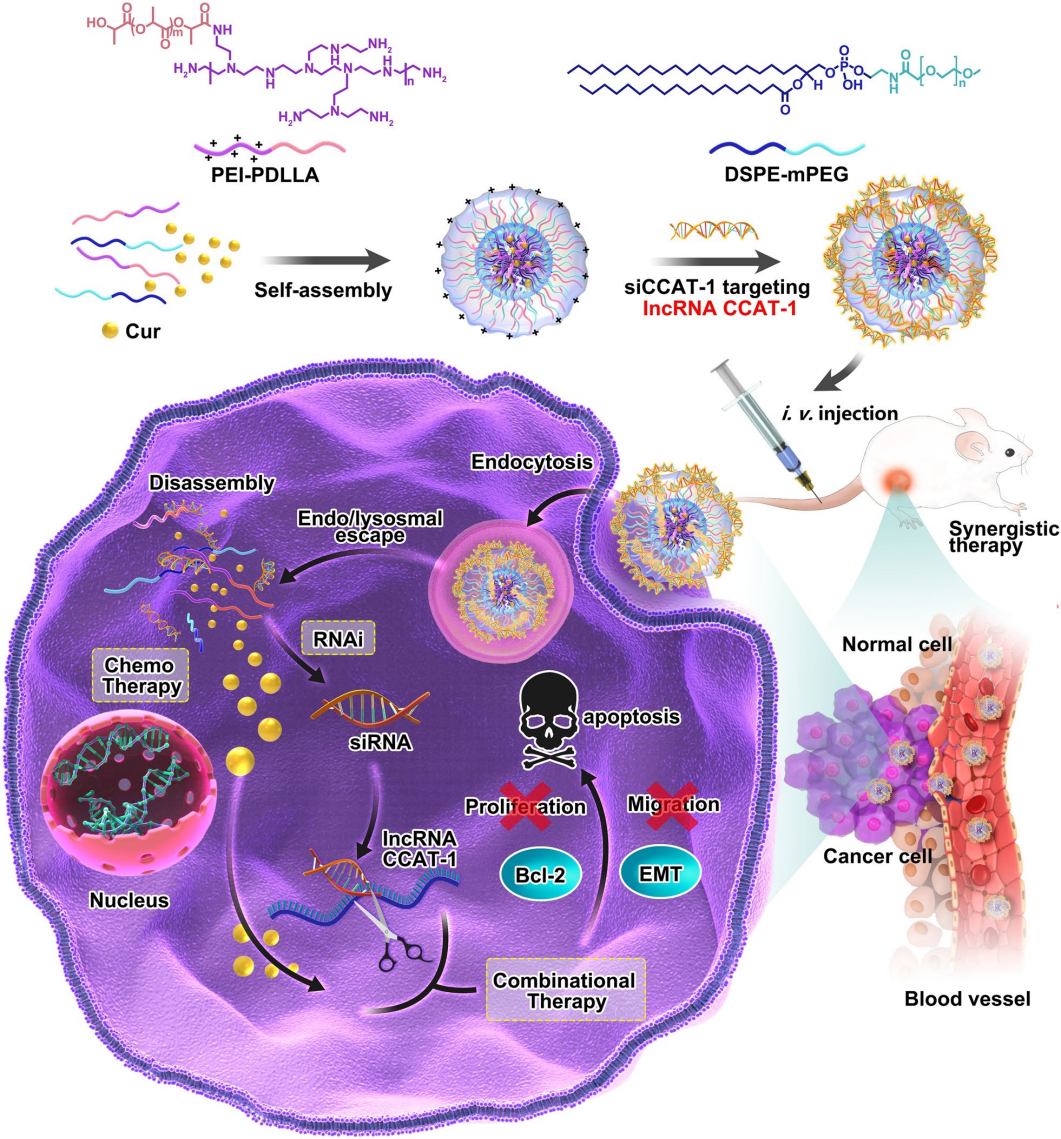
A schematic diagram of amphiphilic copolymer self-assembled micellar system constructed by PEI-PDLLA and DSPE-mPEG for lncRNA CCAT1 silencing and Cur co-delivery. The combinational therapeutic effects were accomplished by regulating multiplex CCAT1-related downstream genes

**Fig. 1:**
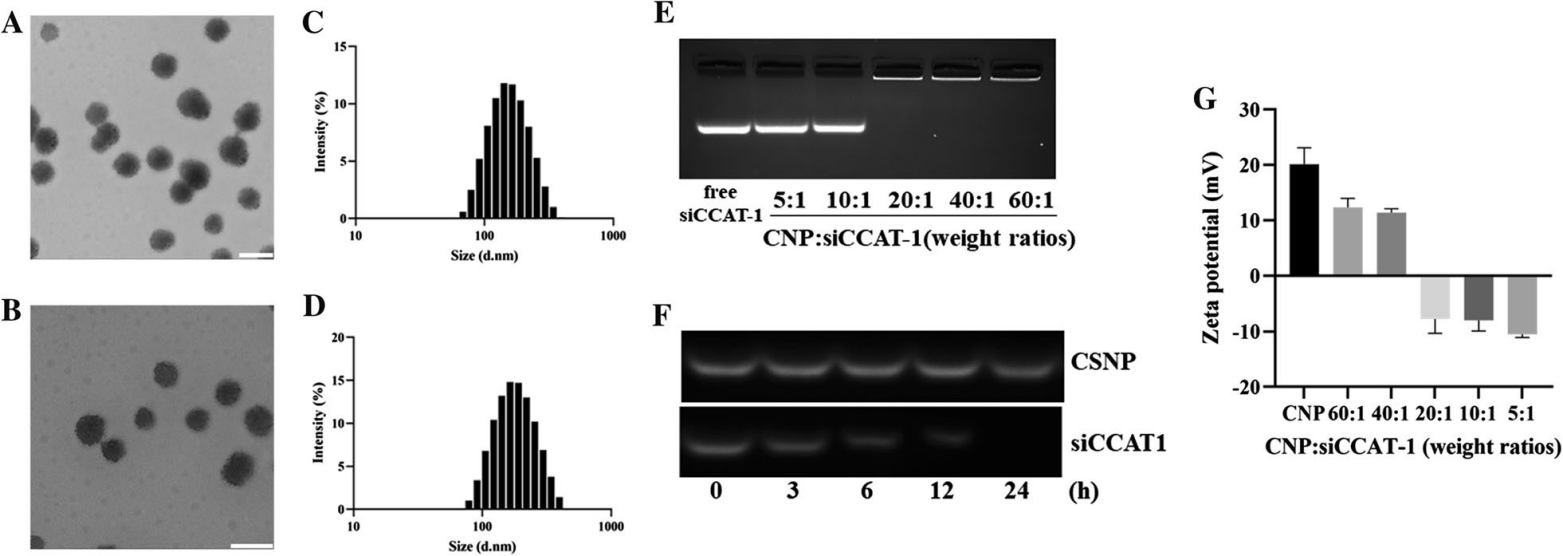
Characterization of the co-delivery system CSNP. **A**, **B** TEM image of CNP and CSNP, respectively. The scale bar was 200 nm. **C**, **D** The particle size distribution of CNP and CSNP, respectively, determined by DLS. **E** Gel electrophoresis retardation assay of CSNP with different weight ratios. **F** siCCAT1 stability in serum environment at extended time points. **G** Zeta potential of CSNP with various CNP:siCCAT1weight ratios

The amphiphilic copolymer PEI-PDLLA was prepared according to the ring-opening polymerization method, which was reported by our laboratory formerly [43]. The preparation procedure of Cur and siCCAT1 co-delivery system CSNP was displayed as Scheme 1. Fourier transform infrared spectroscopy (FT-IR) analysis showed a peak at 1663 cm^−1^ corresponding to PEI, and the strong absorption peak at 1757 cm^−1^ corresponding to PDLLA (Additional file 1: Fig. S1). ^1^H nuclear magnetic resonance (^1^H NMR) in CDCl_3_ as shown in Additional file 1: Fig. S2, the signal at 2.1 ~ 3.5 ppm in PEI-PDLLA assigned to PEI (–NHCH_2_CH_2_–), indicating that the hydrophilic PEI backbone was linked to the hydrophobic PDLLA successfully. To obtain the most appropriate drug loading content (LC) and drug encapsulation efficiency (EE) of Cur and other physicochemical properties for Cur-loaded NP (CNP), which was prepared by film dispersion method, we made a thorough inquiry of the effect of various weight ratio of PEI-PDLLA, DSPE-mPEG and Cur. Comparing to single-component nanoparticles, polymeric hybrid nanoparticles like CSNP formed by self-assembly from multiple amphiphilic copolymers with various effects can realize multifunctionality, such as co-delivery of disparate cargos with opposite properties of hydrophilic and hydrophobic, or zeta potential. It was also controllable for size, zeta potential, EE, and LC of CSNP by changing the mass ratio of components. Firstly, we explored the cytotoxicity of the bare PEI-PDLLA NP to HT-29 cells. As shown in Additional file 1: Fig. S3, the bare PEI-PDLLA NP was toxic to HT-29 cells due to excessive positive charge, especially in high concentration at 48 h. As displayed in Additional file 1: Table S1, DSPE-mPEG was used to decrease the zeta potential of CNP, which aimed to better cell endocytosis and easier siCCAT1 release, and a positive correlation increasing in EE from 85 to 97% was observed, while the average size of CNP increased from 130 to 180 nm. The optimal mass ratio of PEI-PDLLA:DSPE-mPEG:Cur (5:5:1) was selected for the following studies on general considerations of the relatively small size, high zeta potential, EE and LC. The spherical morphology of CNP and CSNP were measured by transmission electronic microscopy (TEM) (Fig. 1A, B), and dynamic light scattering (DLS) analysis established that CNP and CSNP exhibited an equalized distribution with an average size of ∼ 131 and ~ 151 nm furthermore (Fig. 1C, D). In the next step, we explored the ability of CNP to absorb negatively charged siCCAT1 through electrostatic interaction, and according to the obtained results of DLS analysis, the zeta potential of CSNP transformed from + 12.37 mV to − 10.48 mV, while the weight ratio of CNP and siCCAT1 varied from 60:1 to 5:1 (Fig. 1G). To further evaluate whether siCCAT1 could be adsorbed onto CNP successfully, gel electrophoresis retardation assay was implemented with weight ratios of CNP and siCCAT1 ranging from 5:1 to 60:1 (Fig. 1E), whose results showed that the vast majority of siCCAT1 could be blocked by CNP when the mass ratio more than 20:1. These results of DLS analysis and agarose gel electrophoresis assay confirmed that CNP had the ability to bind negatively charged siCCAT1 through electrostatic interaction, and we adopted the 20:1 mass ratio of CNP and siCCAT1 to prepare CSNP in follow-up studies. Additionally, serum stability analysis showed that naked siCCAT1 degraded gradually during 24 h, while the siCCAT1 encapsulated in CSNP undegraded (Fig. 1F), which indicated that CSNP could improve the stability of siCCAT1and protect siCCAT1 from fast degradation in serum. In addition, there was no significant changes in the average size or surface zeta potential of CSNP for 5 days as shown in Additional file 1: Figs. S4 and S5, indicating the CSNP were stable and suitable for further application.

### In vitro self-tracking of cellular uptake and endosomal/lysosomal escape

**Fig. 2:**
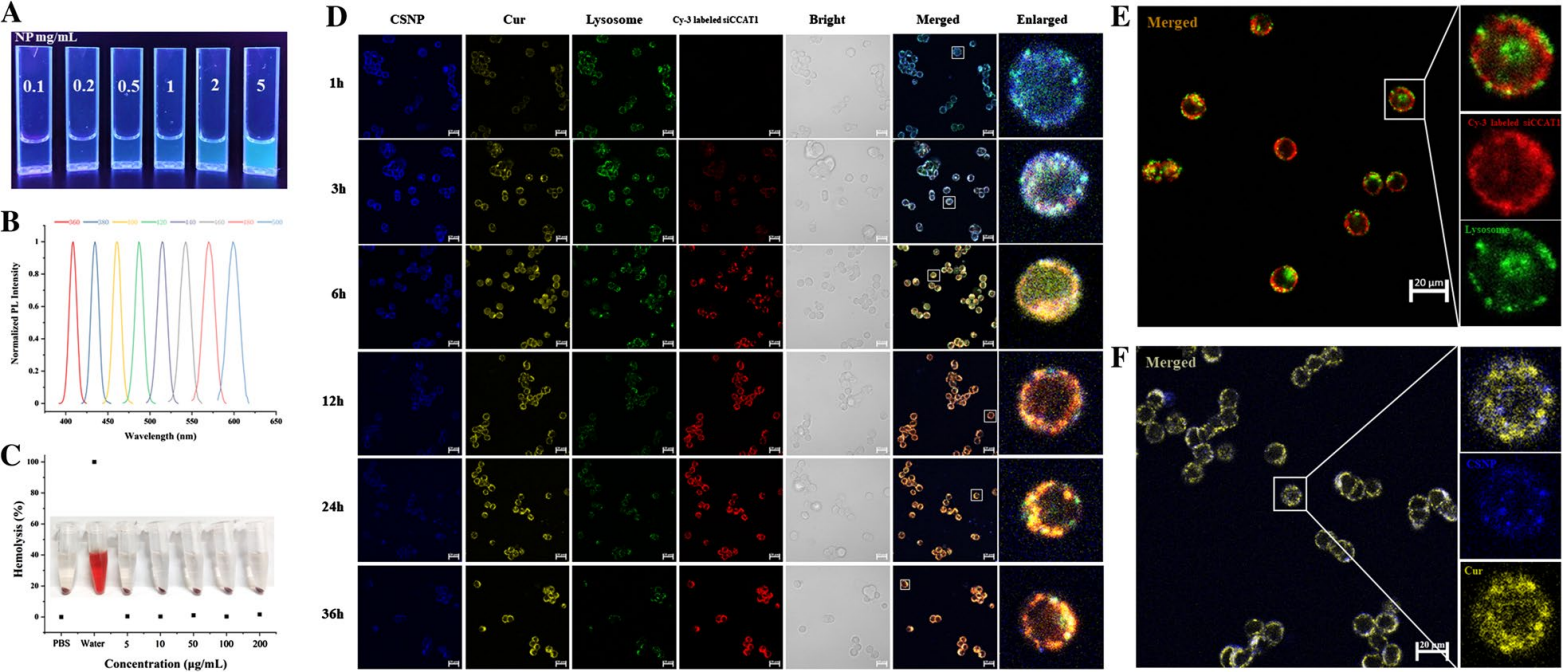
CSNP was used as an in vitro self-tracking agent. **A** Images of NP solutions with a variety of densities token under 365 nm ultraviolet light. **B** Normalized luminescence spectra of the NP. **C** Hemolysis assay of multiple densities of NP. **D** Pictures of HT-29 cells token under CLSM co-cultured with Cur (yellow) and Cy3 (red) labeled siCCAT1 co-loaded CSNP for different hours. The endosome/lysosome was displayed as green and CSNP was displayed as blue. **E** The successful endosomal/lysosomal escape of SNP. **F** The Cur was released from CSNP after 6 h post-incubation. All scale bars were 20 μm

PEI has the property of autofluorescence, which had been widely investigated, and PEI-PDLLA copolymer solution had obviously autofluorescence ability under 365 nm excitation UV light as reported in our previous work with the similarity to the fluorescence emission of PEI [44]. To investigate the fluorescence properties of NP (blank DSPE-mPEG and PEI-PDLLA NP), concentration-dependent autofluorescence and excitation-dependent photoluminescence behavior in the fluorescence spectrum was detected (Fig. 2A, B), which was suitable for bio-imagining at cellular and living level. A major challenge of RNAi is the delivery of RNA. Naked RNA is easily degraded by RNase in plasma and tissues, or cleared by liver and kidney, and recognized by the immune system. The electronegativity and molecular weight of RNA make it difficult to pass through biofilms freely, and it may be retained in the endocytic body and cannot function even after entering through the cell membrane. So, it is important for the delivery system to help RNA achieve endosomal/lysosomal escape. Based on this, to evaluate whether siCCAT1 could effectively escape endosomes/lysosomes at the cellular level, we used Cy3 labeled siCCAT1 to prepare SNP (siCCAT1-loaded NP), and then co-cultured with HT-29 cells. The confocal laser scanning microscopy (CLSM) images (Fig. 2D) showed that clear and strong blue fluorescence of CSNP, most of which coincided with the position of endosomes/lysosomes, which were labeled by LysoTracker Green, only after 1 h post-incubation. It indicated that the CSNP could be endocytosed fast and locate in endosomes/lysosomes, moreover, the NP could act as a particular fluorescent dye for self-tracking in vitro fluorescence imaging. After 4 h, siCCAT1 and endosomes/lysosomes overlapped only a small part, and the red fluorescent signal was mainly distributed in the cytoplasm (Fig. 2E). The signals of Cur and NP separated after 6 h, either (Fig. 2F). The increasing yellow fluorescence of Cur and red fluorescence of Cy3 labeled siCCAT1 were detected, while the blue fluorescence of NP and the green fluorescence of endosomes/lysosomes were faded, which implied that Cur and siCCAT1 might release from CSNP and make endosomes/lysosomes damaged. In addition, we also explored the in vitro release of Cur and siCCAT1 in PBS at pH 7.4 and pH 5.5. As shown in Additional file 1: Fig. S6, in the first 12 h, all Cur and siCCAT1 were released with an initial burst. After that it was followed by a continuously slow-release phase, and finally the amount of Cur and siCCAT1 released in PBS at pH 7.4 was less than that at pH 5.5. In contrast, the release rate of siCCAT1 from CSNP was faster than Cur in PBS at pH 5.5, which reason might be that CSNP was more likely to collapse under acidic conditions and it was difficult to maintain the positive charge for adsorbing siCCAT1. The above results could confirm that CSNP has the ability to help siCCAT1 and Cur realize endosomal/lysosomal escape to the cytoplasm effectively and damage HT-29 cells, which might be attributed to the proton sponge effect of the PEI-PDLLA copolymer.

### In vitro combinational therapeutic anti-cancer effects of CSNP

**Fig. 3:**
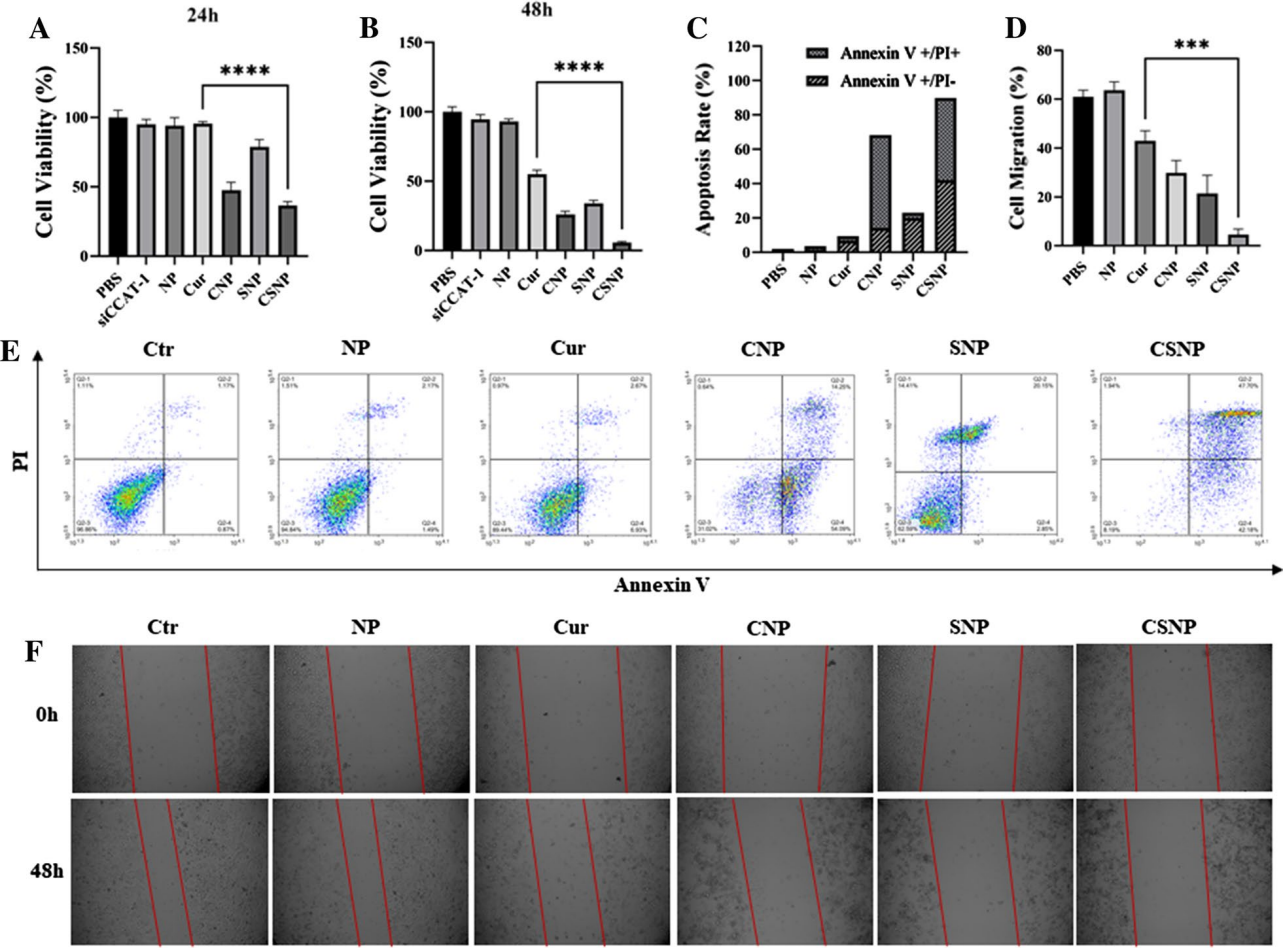
In vitro combinational therapeutic anti-cancer effects of CSNP. **A**, **B** Relative viability of HT-29 cells cultured with different formulations for 24 h and 48 h. NP: blank nanoparticles. Cur: free Cur. **C** Histogram analysis of the FCM analysis. **D** Histogram analysis of wound healing assays. **E** FCM analysis of a variety of preparations treated HT-29 cells. **F** Measured and photographed the wound healing width at 0 h and 48 h

To evaluate the 50% inhibitory concentration (IC_50_) of Cur in HT-29 cells, the concentration of HT-29 cells treated with Cur ranges from 5 μg/mL to 75 μg/mL, and the IC_50_ of Cur to HT-29 cells was about 18 μg/mL at 24 h. In order to study whether CSNP-mediated intracellular co-delivery of Cur and siCCAT1 could realize a synergistic therapeutic effect in HT-29 cells, HT-29 cells were treated with a different formulation of PBS, siCCAT1, NP, free Cur, CNP, SNP, and CSNP at a concentration of 10 μg/mL (lower than that of IC_50_ of Cur), for 24 h or 48 h, and the cytotoxicity of HT-29 cells was evaluated by CCK-8 assay. As depicted in Fig. 3A, B, there was no apparent death of HT-29 cells in the group of NP treated, which indicated the good biocompatibility and biosafety of the NP. Moreover, we used different concentrations of NP, from 10 μg/mL to 200 μg/mL, to co-culture with HT-29 cells for 24 h and 48 h, and there was also no obvious toxicity to HT-29 cells (Additional file 1: Fig. S7), which also confirmed low cytotoxicity of NP. The HT-29 cells co-cultured with SNP showed cell viability reduction, indicating that inhibiting CCAT1 in HT-29 cells could lead to the apoptosis of CRC cells, which was consistent with many reports that CCAT1 could promote tumorigenesis and development [45]. The free Cur, CNP and CSNP were all showed significant cytotoxicity to HT-29 cells, and CNP exhibited higher cytotoxicity than free Cur, indicating that CNP had enhanced the sensitivity of HT-29 cells to the toxicity of Cur. Furthermore, significant decreases in cell viability of CSNP treated groups were observed at 24 h and 48 h, demonstrating the co-delivery of Cur and siCCAT1 by CSNP might notably increase the sensitivity of HT-29 cells to anti-cancer efficiency of Cur and the silencing effect of CCAT1 by combinational therapy.

### In vitro CSNP-mediated enhancement of apoptosis and inhibition of migration

**Fig. 4:**
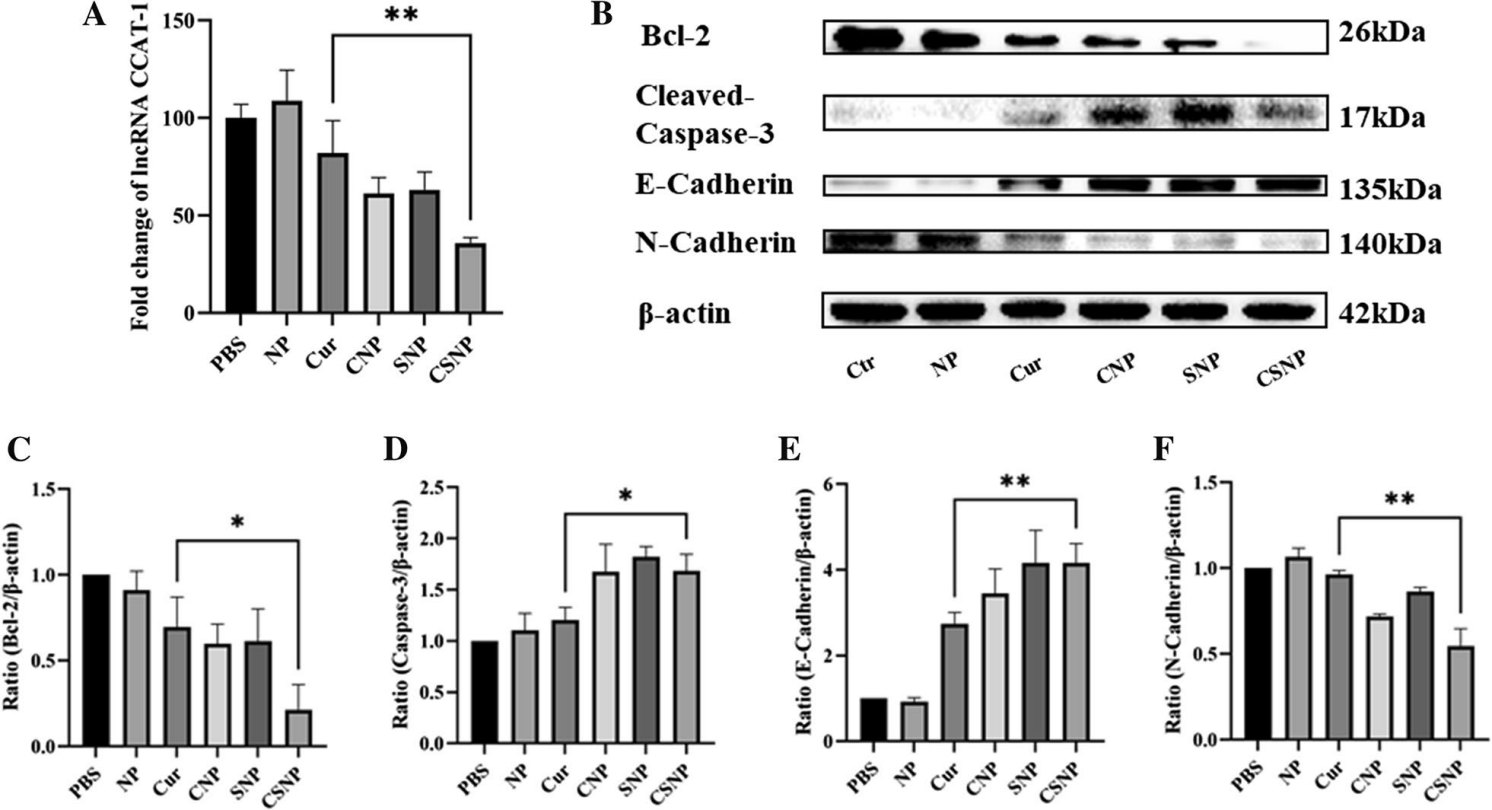
In vitro CSNP-mediated enhancement of apoptosis and inhibition of migration. **A** The levels of lncCCAT1 detected by qRT-PCR assay with different formulations. **B** Western blot analyzed the protein expression content of Bcl-2, Caspase-3, E-Cadherin and N-Cadherin. **C**–**F** Histogram analysis of western blot

A large number of studies had shown that the mitochondrial pathway plays a vital role in the cell survival mechanism. Among many apoptosis-regulating genes, the Bcl-2 protein family and the Caspase family are currently the most concerned [46, 47]. Caspase-3 is the most critical apoptosis executive protease and Bcl-2 is currently known as the most important regulatory gene in the process of apoptosis regulation. Bcl-2 and Caspase-3 had been identified as the major downstream aim of Cur and CCAT1, and down-regulating CCAT1 can inhibit the expression of Bcl-2 gene and promote the expression of Caspase-3 [45, 48–50]. In addition, Bcl-2 has been proved to be an important oncogene, which can regulate the apoptosis of cancer cells. Inhibiting the expression of Bcl-2 can make cancer cells sensitive to chemotherapy, thereby improving the overall therapeutic effect [51, 52]. Since EMT is an effective way for epithelial cells to acquire the ability to migrate, it has become an important way for the infiltration and metastasis of epithelial cell carcinoma, which accounts for more than 90% of malignant tumors [51, 53, 54]. It has been demonstrated that inhibition of CCAT1 regulates the expression of a variety of transcription factors that mediate EMT, such as downregulating N-Cadherin while upregulating E-Cadherin, and the same concept has also been reported in Cur related researches [55–58]. Therefore, further studies were carried out to demonstrate whether CSNP-mediated co-delivery Cur and siCCAT1 could have synergetic enhancement of apoptosis and inhibition of migration. FCM analysis showed the apoptosis rate of HT-29 cells co-cultured with a variety of preparations. As displayed in Fig. 3C, E, comparing with the PBS group, the apoptosis rate of free Cur, CNP and SNP treated groups were ~ 9%, ~ 67%, and ~ 22%, respectively, while the CSNP treated group led to a notable increase in the apoptosis rate (about 88%), which suggested that the co-delivery of Cur and siCCAT1 mediated by CSNP could synergistically increase the apoptosis of HT-29 cells. Besides that, the level of CCAT1 in cells after treatment with different preparations for 24 h was measured by qRT-PCR, which results validated CNP, SNP and CSNP could all downregulate the expression of CCAT1, and co-delivery of Cur and siCCAT1 by synergy could downregulate much more effectively than separate delivery (Fig. 4A). The western blot results showed that the free Cur, CNP, SNP, and CSNP treatment groups significantly downregulated the expression of Bcl-2, and the CSNP treatment group showed the highest inhibition efficiency of Bcl-2, while the NP treatment group showed no significant difference with control (Fig. 4B, C). The result of Caspase-3 expression was opposite to that of Bcl-2 (Fig. 4D), which suggested the CSNP-mediated enhancement of apoptosis on HT-29 cells might be due to the synergistic therapeutic effect of Cur and siCCAT1, through the Bcl-2 and Caspase-3 mediated mitochondrial apoptosis pathway. The micrometastatic relapse is the key reason for the high mortality of CRC. It could be clearly observed from the western blot results that N-Cadherin expression was down-regulated and E-Cadherin was up-regulated in the CNP, SNP, and CSNP treatment groups (Fig. 4E, F), indicating CSNP might inhibit migration of HT-29 cells through EMT pathway. To make a thorough inquiry of the ability of CSNP-mediated inhibition to the migration of HT-29 cells, the wound healing assay was performed. The progress of wound healing was much slower in the HT-29 cells treated with CNP and SNP than that of the HT-29 cells treated with free Cur and NP, and the HT-29 cells treated with CSNP had almost no migration observed (Fig. 3D, F). The obtained results illustrated that the co-delivery of Cur and siCCAT1 mediated by CSNP could significantly enhance Bcl-2-mediated mitochondrial apoptosis and inhibit EMT-mediated migration through a synergistic effect.

### In vivo fluorescence/photoacoustic imaging

**Fig. 5:**
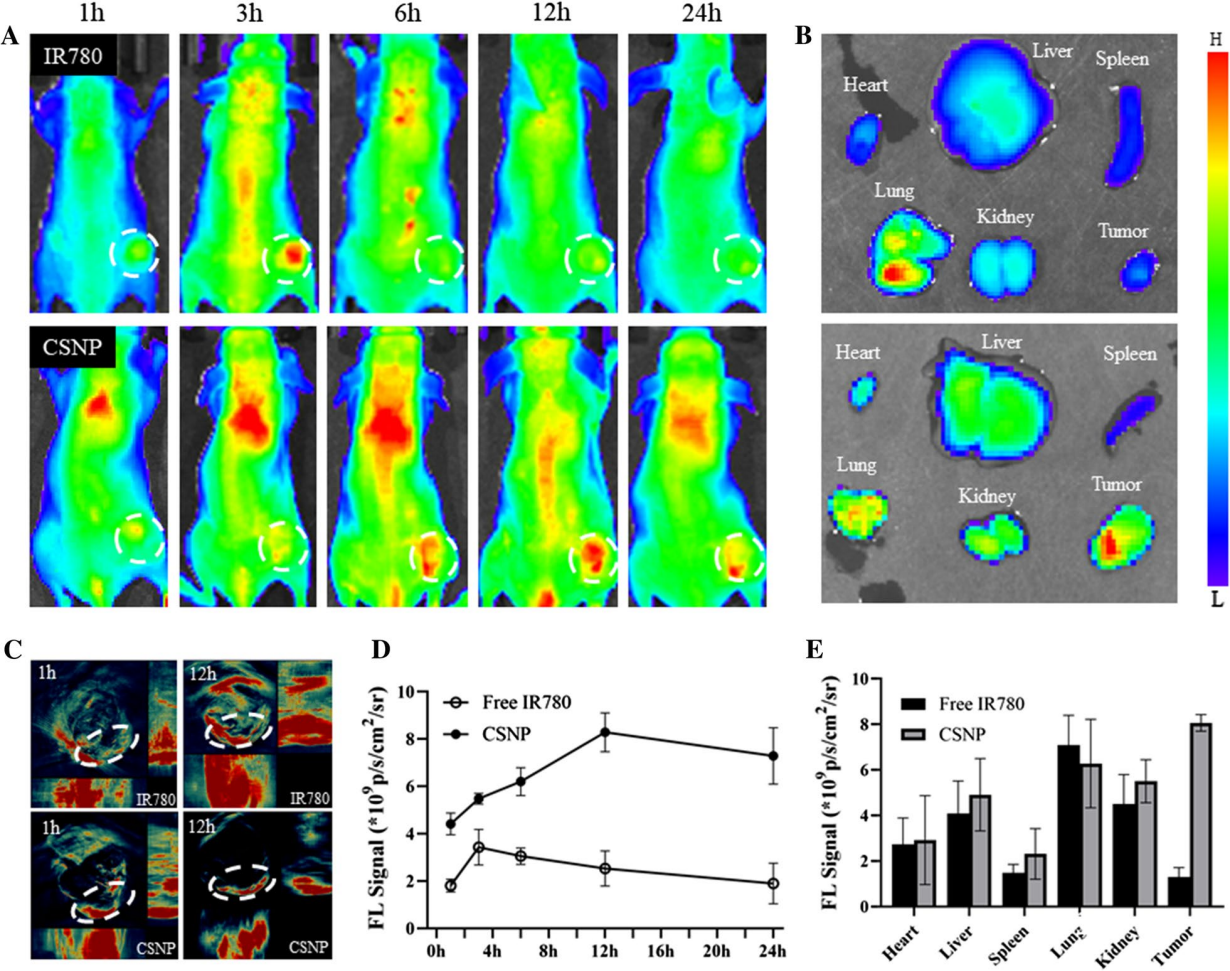
In vivo fluorescence/photoacoustic imaging. **A** The fluorescence photos of the CSNP in HT-29 tumor-bearing nude mice model at different time gaps after free IR780 or CSNP intravenous injection in vivo, and the tumors were highlighted with white circles. **B** Ex vivo fluorescence images of dissected organs and tumors at 24 h post-injection of free IR780 or CSNP. **C** In vivo photoacoustic imaging of HT-29 tumor-bearing nude mice after intravenous injection of free IR780 or CSNP at specific time points. The tumors were highlighted with white circles. **D** Quantitative data (mean ± standard error) of the fluorescence signal (FL signal) emitted from the injection area shown in **A**. **E** Quantitative data (mean ± standard error) of the FL signal emitted from the injection area shown in **B**

The successful in vitro self-tracking results of SNP encouraged us to evaluate tumor-targeting self-tracing capabilities of CSNP in vivo. In order to assess the biosafety of NP in vivo first, a blood compatibility determination was performed. As shown in Fig. 2C, NP did not induce red blood cell hemolysis so far as to a strong density (1 mg/mL), indicating that CSNP had good blood compatibility in vivo. We processed the fluorescence imaging of HT-29 tumor-bearing nude mice with CSNP by intravenous injection and compared them with the free IR780 group. As displayed in Fig. 5A, after the injection intravenously, the fluorescence signal of free IR780 accumulated at the tumor site within 3 h and disappeared quickly at 6 h, while the fluorescence signal strength of the CSNP signal of the tumor site increased progressively and achieved the summit at 12 h. As time goes by, the fluorescence signal of CSNP did not fade until 24 h while the fluorescence signal of free IR780 could only last for about 6 h (Fig. 5D), indicating that CSNP could continuously accumulate in the tumor site through the enhanced permeability and retention effect (EPR). Mice were immolated at 24 h after the injection, and main organs were obtained for ex vivo fluorescence imaging. As displayed in Fig. 5B, strong fluorescence accumulated in the tumor site with the CSNP group, while the fluorescence was mostly enriched in the lungs with the free IR780 group, and the quantification analysis was also consistent with the above results (Fig. 5E). In addition, photoacoustic imaging (Fig. 5C) had the same results as fluorescence imaging, suggesting that CSNP tended to accumulate enrichment at tumor region, and could be used as both fluorescence imaging and photoacoustic imaging agent for self-monitoring.

### In vivo combinational therapeutic anti-cancer effects of CSNP

**Fig. 6:**
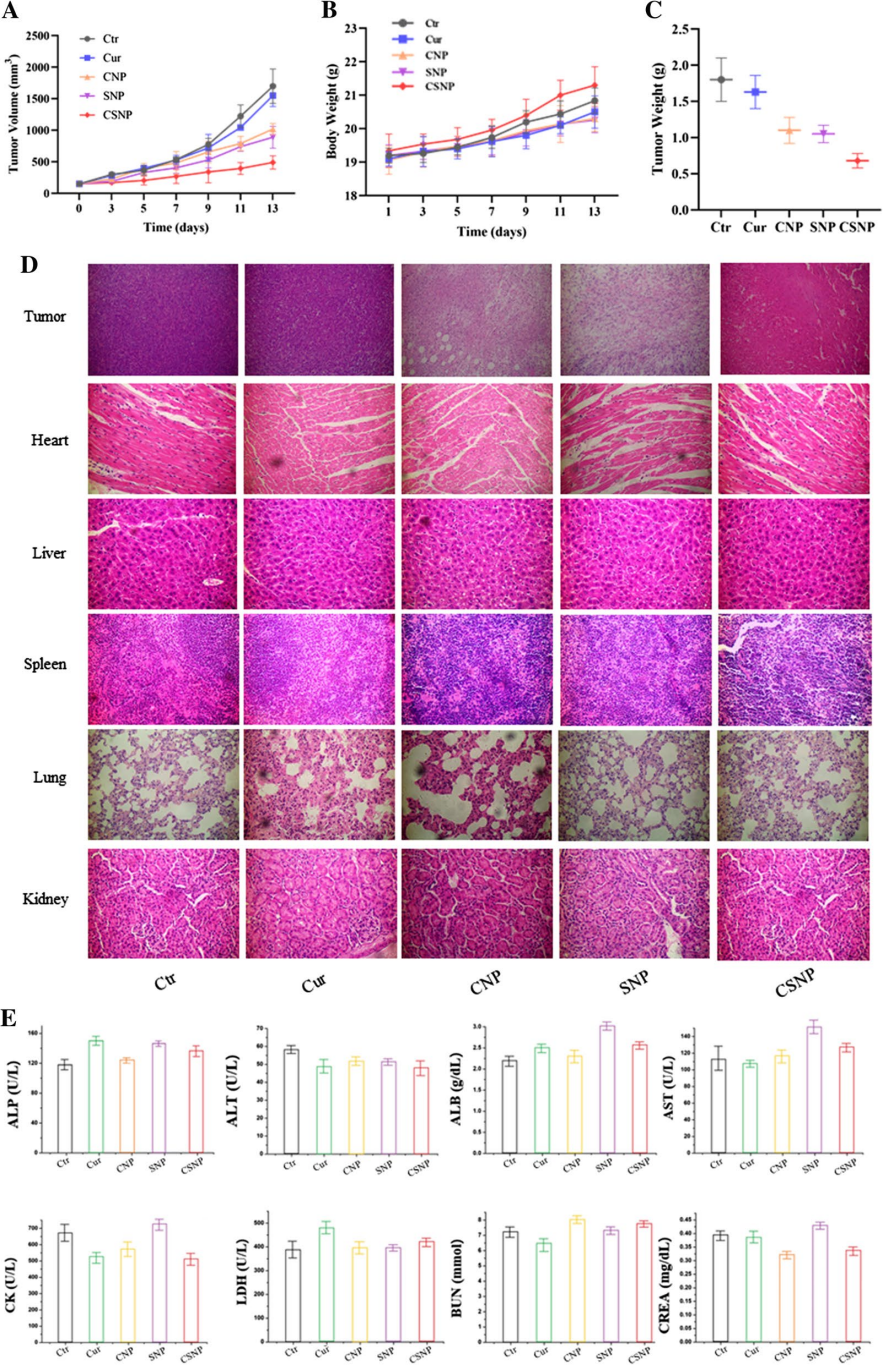
In vivo combinational therapeutic anti-cancer effects of CSNP. After intravenous injection of different preparations, (**A**) the changes of tumor volume and (**B**) body weight of HT-29 tumor-bearing nude mice during treatment, n = 5. **C** Tumor weight of each nude mice after immolated. **D** Histological analysis of tumors and main organs with different treatments. **E** Hemanalysis of ALP, ALT, ALB, AST, CK, LDH, BUN and CREA. *ALP* alkaline phosphatase, *ALT* alanine aminotransferase, *ALB* albumin, *AST* aspartate transaminase, *CK* creatine kinase, *LDH* lactate dehydrogenase, *BUN* blood urea nitrogen, *CREA* creatinine

In order to detect whether NP would damage the tissues of mice during the treatment, we observed mice that were injected with saline and NP for 7 days, and then the main organs were taken for H&E staining. As displayed in Fig. S8, healthy tissues could be observed in the slices, and there were no signs of organ damage. To evaluate combinational therapeutic anti-cancer effects of CSNP in vivo, we established the HT-29 subcutaneous xenografts model. The mice were randomly divided into different treatments and intravenously injected with saline, free Cur, CNP, SNP, and CSNP when the tumor volume of the mice reached about 150 mm^3^. By examining the tumor volume of the mice (Fig. 6A), we found that the control saline group and the free Cur group both showed rapid growth trends, and the CNP group and SNP group had a slight inhibitory effect on tumor growth, while the CSNP group showed a much more superior therapeutic effect than other groups, which implied that the treatment of CSNP had a remarkably advanced activity over free Cur, CNP and SNP, suggesting CSNP-mediated co-delivery of Cur and siCCAT1 had a combinational therapeutic anti-cancer effect through a synergetic function in vivo. In addition, no weight loss was observed in all the treated mice (Fig. 6B), which indicated the co-delivery of Cur and siCCAT1 mediated by CSNP might lead to the expected synergetic therapeutic effect almost without systemic acute toxicity. The mice were sacrificed after the in vivo anti-tumor experiment, and the tumor tissues of the mice were taken out and weighed. Tumor tissue weighing results showed a trend similar to the tumor volume growth curve (Fig. 6C), and the CSNP group also exhibited the best treatment effect. We also performed western blot assay of tumor tissues to explore whether the changes in the content of Caspase-3 and Bcl-2 were consistent with in vitro experiment. As displayed in Additional file 1: Fig. S9, the result was consistent with those of HT-29 cells, which implied CSNP could enhance Bcl-2-mediated mitochondrial apoptosis in vivo by Cur and siCCAT1 co-delivery, too. Hematoxylin–eosin (H&E) staining was used to analyze and examine the histological characteristics of tumors and assess tissue damage of major organs after treatment with various formulations. The results indicated the H&E-dyed profiles of the saline and free Cur treatments both showed the representative shape of large nuclear tumor cells. While the slices of the CNP and SNP treatments showed a decrease in cancer cell density, moreover, the CSNP treatment had minimum cell concentration with contrast. As displayed in Fig. 6D, E, no obvious tissue damage was found under different treatments, and the levels of various serum biochemical indicators were the same as those of the mice in the saline group, and there was no statistical difference among these treatment groups, implying that our Cur-siCCAT1 co-delivery system CSNP had good biosafety and biocompatibility during the treatment.

## Conclusion

In summary, we used the amphiphilic copolymers PEI-PDLLA and DSPE-mPEG to prepare CSNP, which was capable of co-delivery of Cur and siCCAT1 through film dispersion technique and electrostatic interaction. CSNP-mediated Cur and siCCAT1 co-delivery could effectively silence CCAT1 and achieve a synergistic effect, thereby increasing Bcl-2-mediated apoptosis of HT-29 cells, inhibiting EMT-mediated migration of HT-29 cells, and triggering conspicuous anti-tumor efficacy in vivo through combinational therapy with good biocompatibility during the treatment. CSNP could successfully achieve endosomal/lysosomal escape and be well enriched at the tumor site, moreover, be used as an imaging agent for self-tracking fluorescence imaging and photoacoustic imaging at the cellular and living level in a real-time manner. Since the co-delivery of siCCAT1 and anti-cancer drug Cur had the advantage of simultaneously inhibiting tumor growth and migration, CSNP revealed great potential in anti-cancer treatment as a combinational therapeutic strategy in CRC.

## Materials and methods

### Materials

Branched polyethyleneimine (PEI, 25 kDa) was obtained from Sigma-Aldrich (MO, USA). d, l-lactide (DLLA) was obtained from Alfa Aesar (MA, USA). The anhydrous dimethyl sulfoxide (DMSO) was purchased from J&K Scientific Ltd. 1,2-Distearoyl-sn-glycero-3-phosphoethanolamine-*N*-[methoxy(polyethyleneglycol)2000] (DSPE-mPEG) was obtained from A.V.T. (Shanghai) Pharmaceutical Co., Ltd. Curcumin (Cur) was purchased from Alfa Aesar (MA, USA). The 0.8 μm polycarbonate membrane was purchased from Millipore Co. (MA, USA). The mimics of siCCAT1 or cyanine-3 (Cy3) labeled siCCAT1 were obtained from RiboBio Co. (Guangzhou, China). Antibodies of western bolt were purchased from Cell Signaling Technology, Inc and Abcam plc. RNase-free deionized water was purchased from TIANGEN Biotech Co. Ltd (Beijing). LysoTracker Green was obtained from Molecular Probes Inc. (Eugene, OR). The cell counting kit-8 (CCK-8) was obtained from Dojindo Molecular Technologies, Inc., (Japan). Fetal bovine serum (FBS) was obtained from Wisent Inc. 0.25% trypsin–EDTA, RPMI 1640 medium and penicillin/streptomycin were obtained from Thermo Fisher Scientific (MA, USA). The water used was of ultrapure grade and was supplied by a Milli-Q purification system of Millipore Co. (MA, USA).

### Synthesis of PEI-PDLLA copolymers

Pre-dehydrated 15 g of DLLA and 250 mg of PEI were dissolved in 50 mL of anhydrous DMSO. Then 0.05 mol of trimethylamine was added into above solution, and kept the solution stirring under nitrogen for 12 h at 86℃. The reaction solution was then poured into ice water, and the precipitate was collected and washed by distilled water. Finally, the precipitate was dried in a vacuum oven. The chemical structure was characterized by Fourier transform infrared spectroscopy (Spotlight 200i; PerkinElmer, USA) and proton magnetic resonance spectroscopy (AVANCE III HD 400; Bruker, USA).

### Preparation of Cur-siCCAT1 co-loaded nanoparticles (CSNP)

Amounts of PEI-PDLLA, DSPE-mPEG and Cur were dissolved into dichloromethane (DCM) to form 5 mg/mL, 5 mg/mL and 10 mg/mL solutions, respectively. The blank nanoparticles (NP) were obtained by mixing PEI-PDLLA and DSPE-mPEG solutions in various ratios and then rotating evaporation by film dispersion method. Briefly, after mixing the two solutions, a thin film was formed in the round-bottomed flask by a rotary evaporator, and then distilled water was added to form NP by ultrasonic vibration for 0.5 h. The mixtures were prepared with PEI-PDLLA: DSPE-mPEG mass ratios of 5:5, 5:10, 5:20, 10:10 and 10:20.

The Cur-loaded NP (CNP) with different mass ratios of PEI-PDLLA, DSPE-mPEG and Cur was constructed by mixing Cur solution with PEI-PDLLA and DSPE-mPEG solutions together, and then prepared with film dispersion technique as described above. Separation of unloaded Cur by filtering the suspension through a 0.8 μm polycarbonate membrane. The siCCAT1-loaded NP (SNP) was formed by adding different mass ratios of siCCAT1 to NP through electrostatic interaction. The mixture of NP and dissolved siCCAT1 were incubated for 30 min at 25 °C. The Cur-siCCAT1-co-loaded NP (CSNP) was prepared by adding different mass ratios of siCCAT1 to CNP.

### Physicochemical characterization

The average size and zeta potential were detected by dynamic light scattering (DLS) using ZetaSizer Nano series Nano-ZS (Malvern Instruments Ltd, Malvern, UK). After the sample was properly diluted in distilled water, three replicates were analyzed per batch. The morphology of CNP was determined by transmission electron microscopy (TEM) (Ht-7700; HITACHI, Japan). The absorption spectra were analyzed by a UV–vis spectrophotometer (TU-1810; PERSEE, China) and the fluorescence spectra were recorded on a EnSpire^®^ Multimode Plate Reader (PerkinElmer, Fremont, CA, USA).

A UV–Vis spectrophotometer (TU-1810; PERSEE, China) was used to measure the encapsulation efficiency (EE) and load capacity (LC) of Cur at 425 nm. EE and LC were computed by the following formulas:

$${\text{EE}}\% \, = \,\left( {\text{weight of loaded drug}} \right)/\left( {\text{weight of originally added drug}} \right)\, \times \,{1}00\% .$$

$${\text{LC}}\% \, = \,\left( {\text{weight of loaded drug}} \right)/\left( {\text{total weight of nanoparticles and drug}} \right)\, \times \,{1}00\% .$$

### Gel electrophoresis and serum stability assay

CSNP was analyzed by 4% agarose gel electrophoresis. The 4% agarose gel was prepared with tris–acetate-ethylenediaminetetraacetic acid (TAE) buffer containing 0.5 μg/mL Genecolor (Gene-bio, China). For electrophoretic mobility analysis, incubated the sample for 30 min at room temperature, and then 10% glycerol was added to the samples. The gel electrophoresis was conducted at 110 V for 10 min, and then the gel was photographed using a Bio-Rad ChemiDoc™ XRSTouch Imaging System (CA, USA).

For the determination of serum degradation, the naked siCCAT1 aqueous solution and CSNP samples were mixed with FBS, at a ratio of 1:1 to obtain a serum concentration of 50%. The mixtures were then incubated at 37 ℃ for the indicated times. The mixtures were taken out at indicated time interval and then mixed with 2% sodium dodecyl sulfonate (SDS), and which were further loaded onto a 4% agarose gel for gel electrophoresis assay.

### In vitro self-tracking of cellular uptake and endosomal/lysosomal escape

The CRC cells HT-29 were cultured in RPMI 1640 medium containing 10% (v/v) FBS and 1% penicillin/streptomycin (P/S) at 37 ℃ in a humidified atmosphere with 5% CO_2_. HT-29 cells were cultured onto a glass bottom dish, reaching a suitable concentration of 2 × 10^5^ cells per well. The medium was replaced by fresh medium compromising SNP for incubation 0.5 h, 1 h, 2 h, 4 h, 6 h, respectively. After that, HT-29 cells were washed 3 times with PBS, and then stained endosome/lysosome with LysoTracker Green. And cell uptake and endosomal/lysosomal escape of SNP (blue fluorescence) carrying Cy3 labeled siCCAT1 (red fluorescence) and LysoTracker Green labeled endosome/lysosome (green fluorescence) was detected by confocal laser scanning microscope (CLSM, Z-760; Carl Zeiss, Germany).

### In vitro cytotoxicity assays

HT-29 cells were seeded in 96-well plates at a density of 5 × 10^3^ cells per well and cultured for 24 h before subsequent studies. Replaced the current medium with 100 μL of RPMI 1640 containing different equivalent density of PBS, naked siCCAT1, NP, free Cur, CNP, SNP and CSNP for 24 h or 48 h (different preparations containing an equivalent 10 μg/mL of Cur or 100 nM of siCCAT1). The cell viability was estimated using the CCK-8 assay according to manufacturer’s instructions (Dojindo, Japan).

To calculate 50% inhibitory concentration (IC_50_), HT-29 cells in 96-well plates were co-cultured with free Cur in concentrations ranging from 5 to 75 μg/mL. The IC_50_ was reckoned based on the dose of Cur that caused 50% cell death compared to the PBS control.

### Cell apoptosis assay

FITC Annexin V/Propidium Iodide (PI) double staining method was used to determine the ability of inducing apoptosis by different treatment. HT-29 cells co-cultured with a variety of NP, free Cur, CNP, SNP and CSNP were evaluated. After 24 h of incubation, by centrifuging at 500 g for 5 min to harvest cells. The Annexin V/PI Apoptosis Detection Kit was used in accordance with the manufacturer’s protocol and subjected to flow cytometry analysis (FCM).

### Western blot assay

Total protein samples in HT-29 cells were extracted with RIPA lysis buffer (Beyotime Biotechnology, Shanghai, China), and then the protein concentration was detected using BCA kit (Beyotime Biotechnology, Shanghai, China). The same amount protein samples were separated by 10% sodium dodecyl sulfate polyacrylamide gel electrophoresis (SDS-PAGE). After transferring it to PVDF membrane (Millipore, USA), the protein was incubated in blocking solution (5% skim milk powder) at room temperature for 1 h. The primary antibody β-actin, Bcl-2, Caspase-3, E-Cadherin and N-Cadherin were diluted as 1: 2000 and incubated with the sample at 4 °C overnight, and then reacted with the secondary antibody labeled with horseradish peroxidase for 1 h. The Bio-Rad ChemiDoc™ Touch Imaging System was used to determine protein levels in cells, with β-actin as an internal reference.

### In vitro wound healing assay

HT-29 cells were seeded in 24-well plates at a density of 2 × 10^5^ cells per well and cultured for 24 h before subsequent studies. Using the tip of 200 µL sterile micropipette scraped the culture monolayer, and keeping the tip perpendicular to the bottom of the petri dish. The cultured cells were incubated with NP, free Cur, CNP, SNP and CSNP for 48 h for the cells repairing the wound. Finally, in order to calculate the mobility, the quantization width in the case where the wound was observed through a microscope.

### In vivo imaging

The female BALB/c nude mice were obtained from Vital River Laboratory Animal Technology Co., Ltd. (Beijing, China). All relevant experiments were carried out following the ethical rules enacted by Experimental Animal Ethics Committee in Beijing. CSNP and free IR780 were intravenously injected into tumor-bearing nude mice, and then the IVIS Spectrum in vivo optical imaging system (PerkinElmer, USA) and multispectral optical tomography system (MSOT invasion 128; iThera medical, Germany) were used for fluorescence and photoacoustic imaging at specific time points (1 h, 3 h, 6 h, 12 h, 24 h), respectively. All mice were immolated after imaging, and tumors and major organs were gathered for ex vivo fluorescence imaging further.

### In vivo antitumor assessment

To establish a tumor model, 1 × 10^6^ HT-29 cells dispersed in 100 μL of PBS were injected subcutaneously into the right hind limb of mice, which were divided into 5 groups (n = 5 per group) stochastically when the tumor volumes grown into ~ 150 mm^3^, and saline, free Cur, CNP, SNP and CSNP were injected through the tail vein (Cur 5 mg/kg, siCCAT1 2 mg/kg) every 2 days, respectively. The tumor volume and body weight of the experimental mice were measured every other day. After all mice were immolated on the 14th day, and tumors were excised and weighed.

### Statistical analysis

All experiments were independently repeated at least 3 times (n = 3), unless otherwise stated. Data were expressed as mean ± SD. To evaluate the significance of the difference between the treatment groups Student’s t-test was used, and the statistical significance was *p < 0.05, **p < 0.01, *** p < 0.005 and ****p < 0.001.

## Supplementary Information

**Additional file 1: Scheme S1.** (A) Synthesis route of PEI-PDLLA and (B) The structure of DSPE–mPEG2000. **Fig. S1**. FT-IR spectrum of PEI, PDLLA and PEI-PDLLA. **Fig. S2**. (A) ^1^H NMR spectrum of PEI, PDLLA and PEI-PDLLA (The deuterated chloroform (CDCl_3_) was used as solvent), and (B) partial amplification. **Fig. S3**. Cytotoxicity of PEI-PDLLA blank micelles. **Fig. S4**. The average size of CSNP after maintained in PBS for different time intervals. **Fig. S5**. Zeta potential of CSNP in PBS (pH = 7.4) at different time intervals. **Fig. S6**. In vitro Cur and siCCAT1 release profiles from CSNP in PBS at pH 5.5 and pH 7.4 at 37 ℃, respectively. **Fig. S7**. Cytotoxicity of PEI-PDLLA/DSPE-mPEG blank micelles. Fig. S8. Histological analysis of main organs with saline and NP treatments, respectively. **Fig. S9**. The protein expression level of Bcl-2 and Caspase-3 of tumor tissues detected by western blot assay. **Table. S1**. The influences of formulation parameters on the size, zeta potential and Cur drug encapsulation efficiency (EE) and loading content (LC).

## Abbreviations

CCAT1: Colon cancer-associated transcript-1
lncRNA: Long noncoding RNA
CRC: Colorectal cancer
PEI-PDLLA: Polyethyleneimine-poly (d, l-lactide)
DSPE-mPEG: 1,2-Distearoyl-sn-glycero-3-phosphoethanolamine-*N*-[methoxy (polyethylene glycol)]
siCCAT1: Small interfering RNA targeting lncRNA CCAT1
Cur: Curcumin
NP: The blank nanoparticles were obtained by mixing PEI-PDLLA and DSPE-mPEG solutions through film dispersion method
CNP: The Cur-loaded NP
SNP: The siCCAT1-loaded NP
CSNP: The Cur-siCCAT1-co-loaded NP
IR780: 2-[2-[2-Chloro-3-[(1,3-dihydro-3,3-dimethyl-1-propyl-2*H*-indol-2-ylidene)ethylidene]-1-cyclohexen-1-yl]ethenyl]-3,3-dimethyl-1-propylindoliumiodide

## Declarations

### Availability of data and materials

Without restrictions.
