# Supplementary material for: Self-assembled fluorescent hybrid nanoparticles-mediated collaborative lncRNA CCAT1 silencing and curcumin delivery for synchronous colorectal cancer theranostics
Source: J Nanobiotechnology. 2021 Aug 11;19:238. doi: 10.1186/s12951-021-00981-7 (PMC8359047; doi:10.1186/s12951-021-00981-7)
Supplement: Supplementary file 1 — Additional file 1: Scheme S1. (A) Synthesis route of PEI-PDLLA and (B) The structure of DSPE–mPEG2000. Fig. S1. FT-IR spectrum of PEI, PDLLA and PEI-PDLLA. Fig. S2. (A) 1H NMR spectrum of PEI, PDLLA and PEI-PDLLA (The deuterated chloroform (CDCl3) was used as solvent), and (B) partial amplification. Fig. S3. Cytotoxicity of PEI-PDLLA blank micelles. Fig. S4. The average size of CSNP after maintained in PBS for different time intervals. Fig. S5. Zeta potential of CSNP in PBS (pH = 7.4) at different time intervals. Fig. S6. In vitro Cur and siCCAT1 release profiles from CSNP in PBS at pH 5.5 and pH 7.4 at 37 ℃, respectively. Fig. S7. Cytotoxicity of PEI-PDLLA/DSPE-mPEG blank micelles. Fig. S8. Histological analysis of main organs with saline and NP treatments, respectively. Fig. S9. The protein expression level of Bcl-2 and Caspase-3 of tumor tissues detected by western blot assay. Table. S1. The influences of formulation parameters on the size, zeta potential and Cur drug encapsulation efficiency (EE) and loading content (LC). [file 12951_2021_981_MOESM1_ESM.docx]

Self-assembled fluorescent PEI-PDLLA/DSPE-mPEG hybrid micelles-mediated collaborative lncRNA CCAT1 silencing and curcumin delivery for synchronous colorectal cancer theranostics

Fan Jia^a,c,1^ ,Yunhao Li^b,1^, Xiongwei Deng^a,^* , Xuan Wang^a,c^, Xinyue Cui^a^, Jianqing Lu^a,^*, Zian Pan ^a,c^ ,Yan Wu^a,c,^*

^a^*CAS Key Laboratory for Biomedical Effects of Nanomaterials and Nanosafety, CAS Center for Excellence in Nanoscience, National Center for Nanoscience and Technology, Beijing 100190, China*

^b^*Department of General Surgery, Peking Union Medical College Hospital, Peking Union Medical College, Chinese Academy of Medical Sciences, Beijing 100730, P. R. China*

^c^*University of Chinese Academy of Sciences, Beijing 100049, P. R. China*

^*^Corresponding author at: CAS Key Laboratory for Biomedical Effects of Nanomaterials and Nanosafety, CAS Center for Excellence in Nanoscience, National Center for Nanoscience and Technology, Beijing 100190, China

*E-mail addresses*: [dengxiongwei.happy@163.com](mailto:dengxiongwei.happy@163.com) (X. Deng), [lujq@nanoctr.cn](mailto:lujq@nanoctr.cn(J) (J. Lu), wuy@nanoctr.cn (Y. Wu)

^1^Fan Jia and Yunhao Li contributed equally to this work.


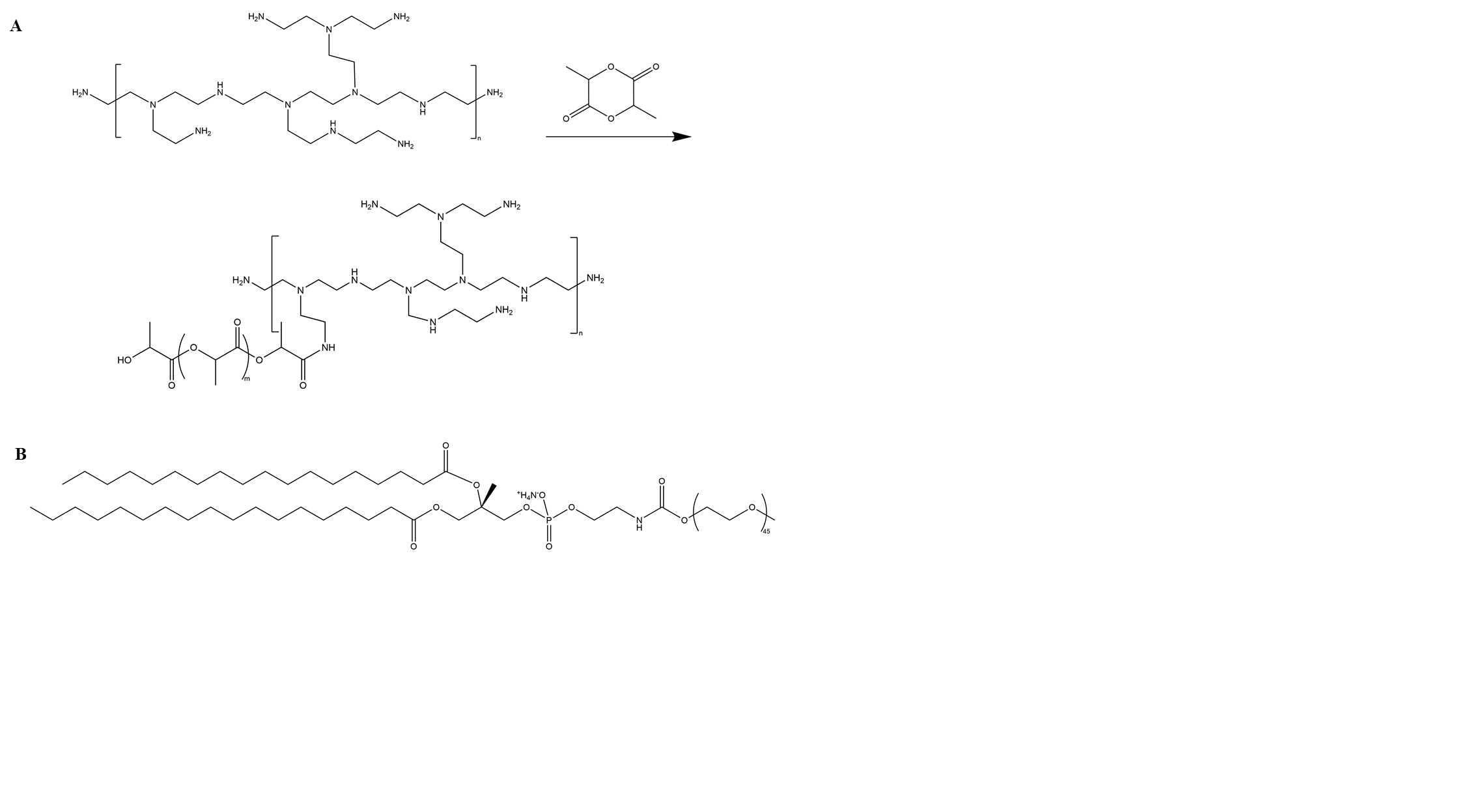


Scheme. S1. (A) Synthesis route of PEI-PDLLA and (B) The structure of DSPE–mPEG2000.


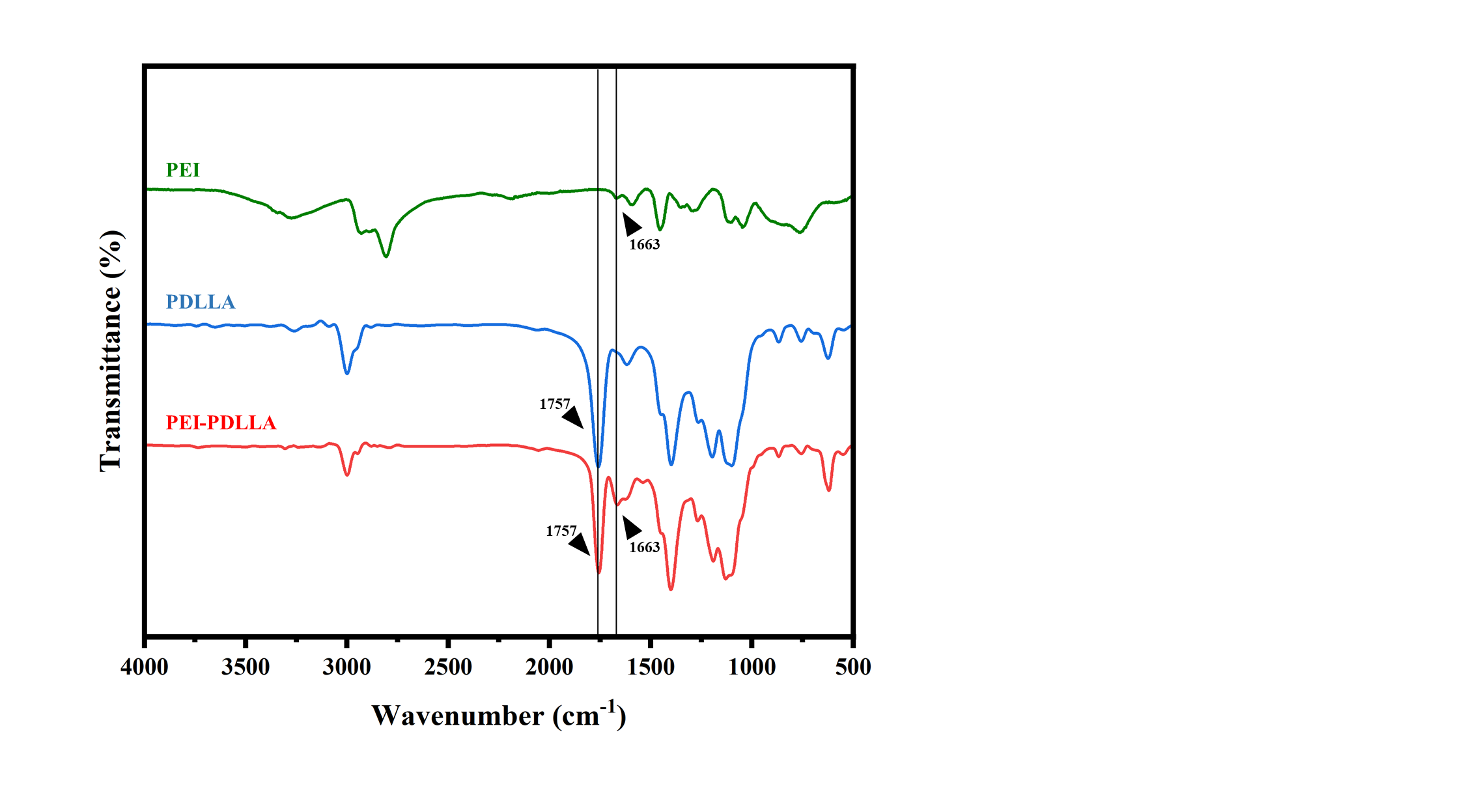


Fig. S1. FT-IR spectrum of PEI, PDLLA and PEI-PDLLA.


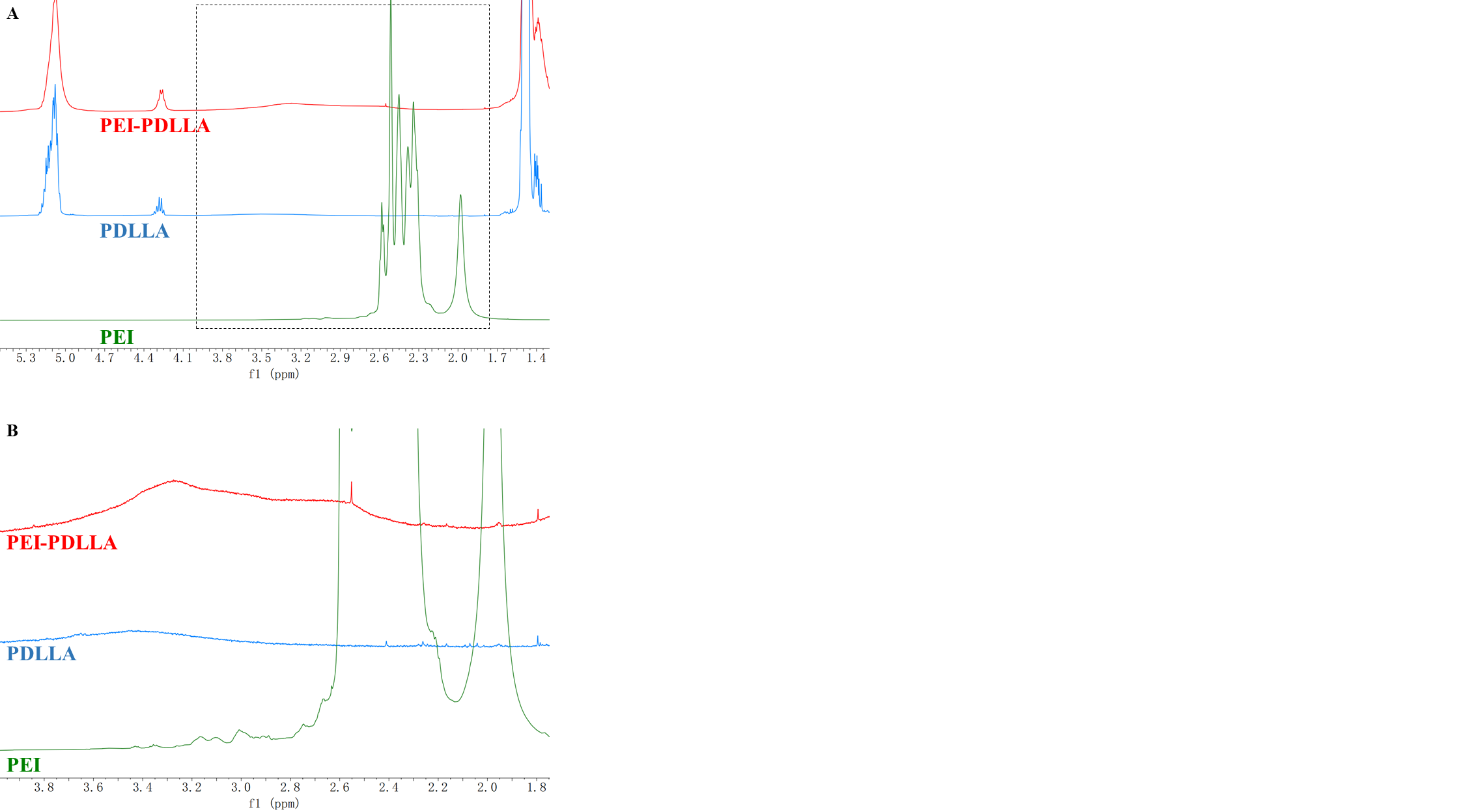


Fig. S2. (A) ^1^H NMR spectrum of PEI, PDLLA and PEI-PDLLA (The deuterated chloroform (CDCl_3_) was used as solvent), and (B) partial amplification.


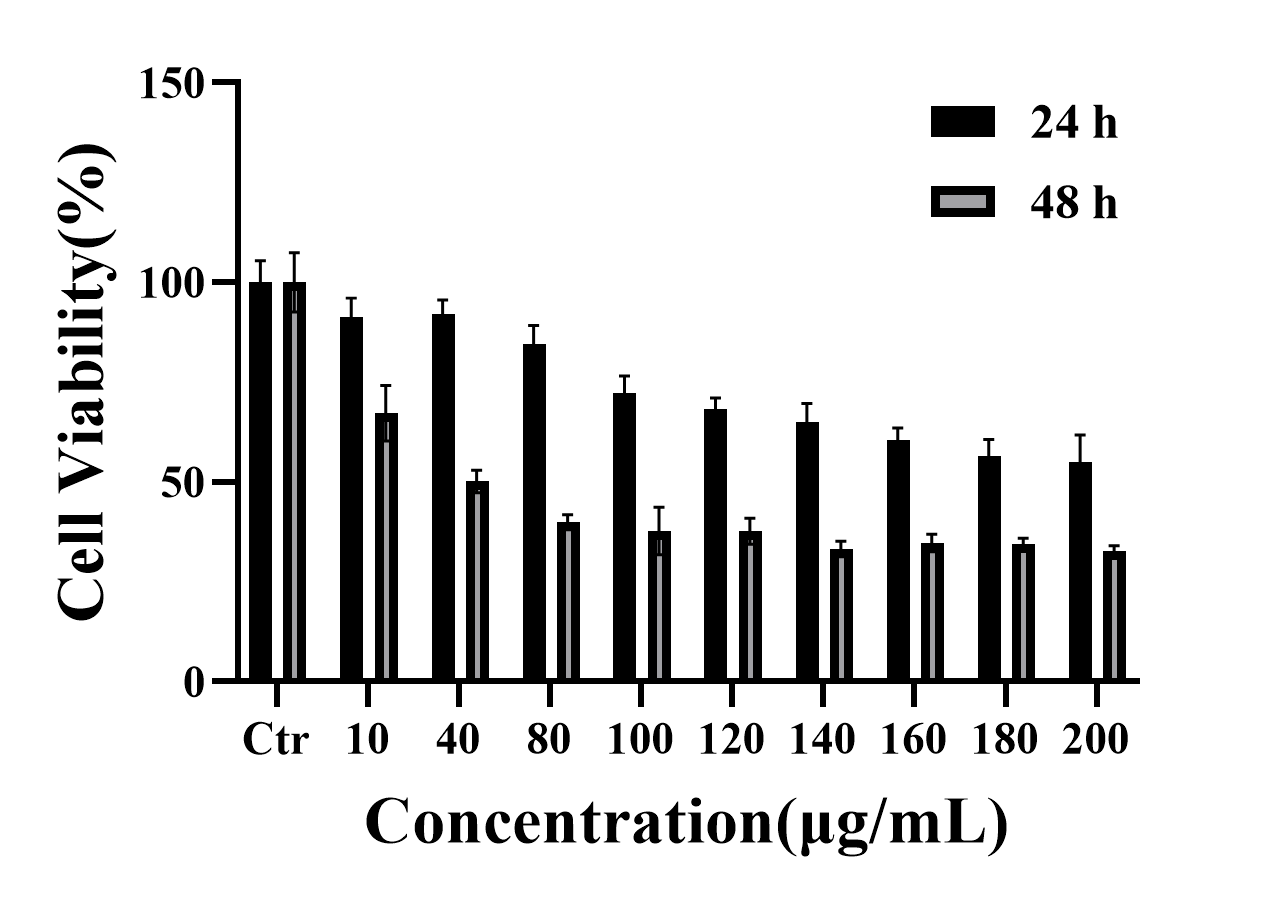


Fig. S3. Cytotoxicity of PEI-PDLLA blank micelles.


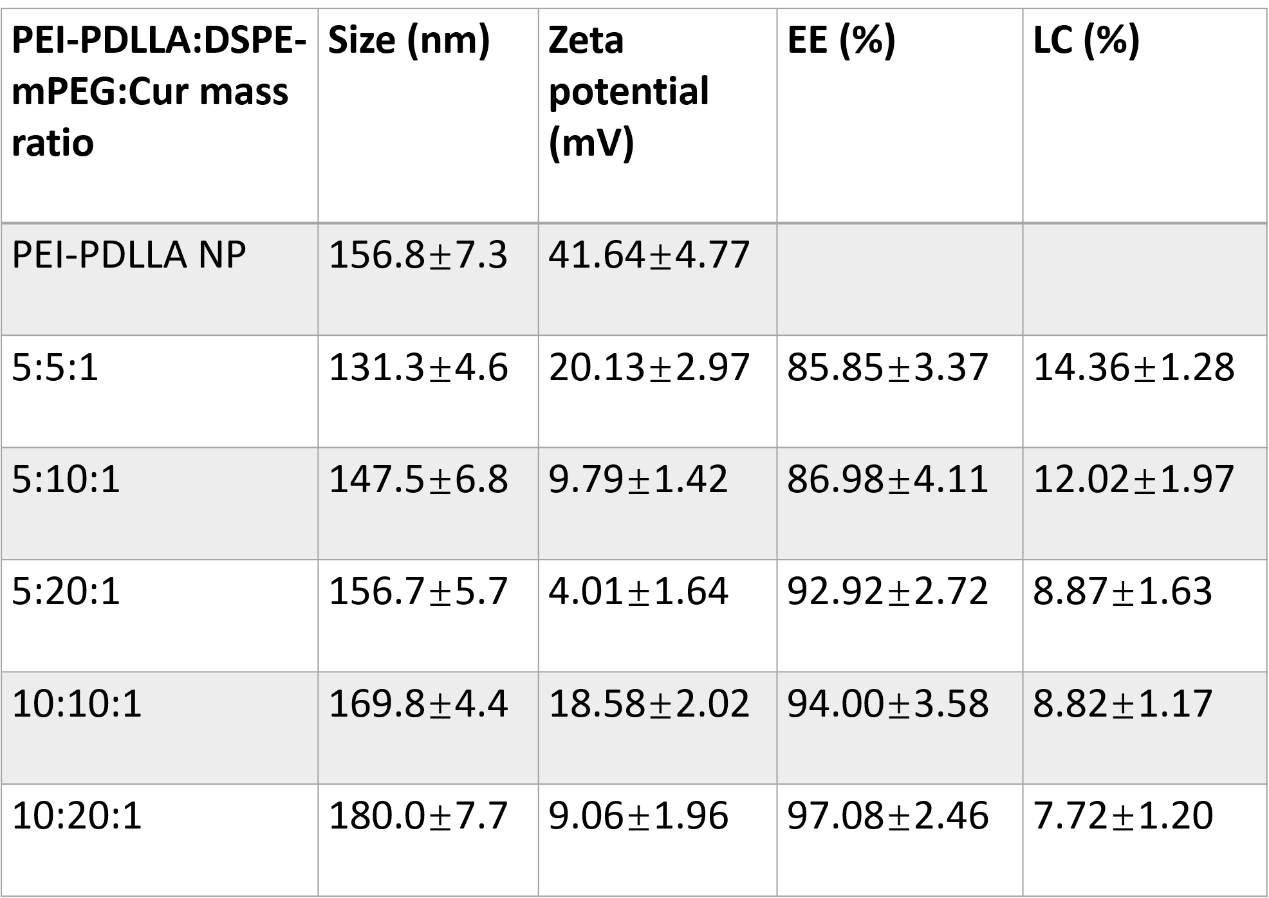


Table. S1. The influences of formulation parameters on the size, zeta potential and Cur drug encapsulation efficiency (EE) and loading content (LC).


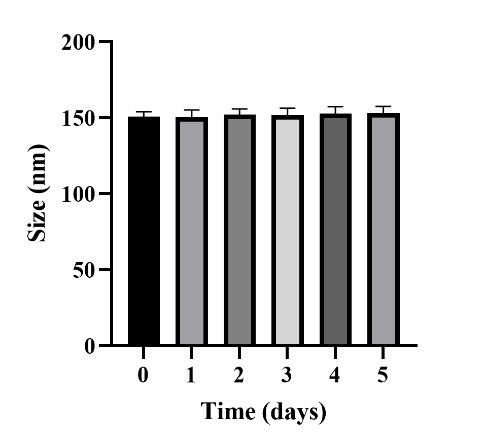


Fig. S4. The average size of CSNP after maintained in PBS for different time intervals.


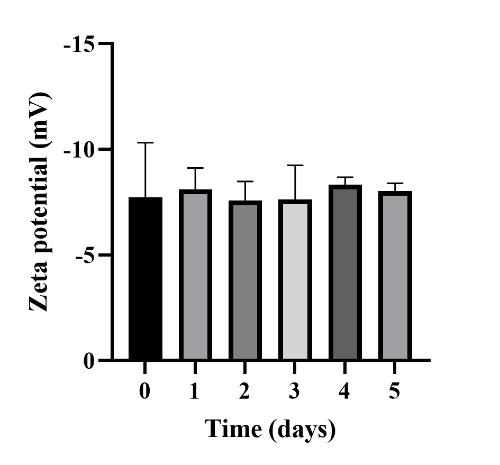


Fig. S5. Zeta potential of CSNP in PBS (pH = 7.4) at different time intervals.


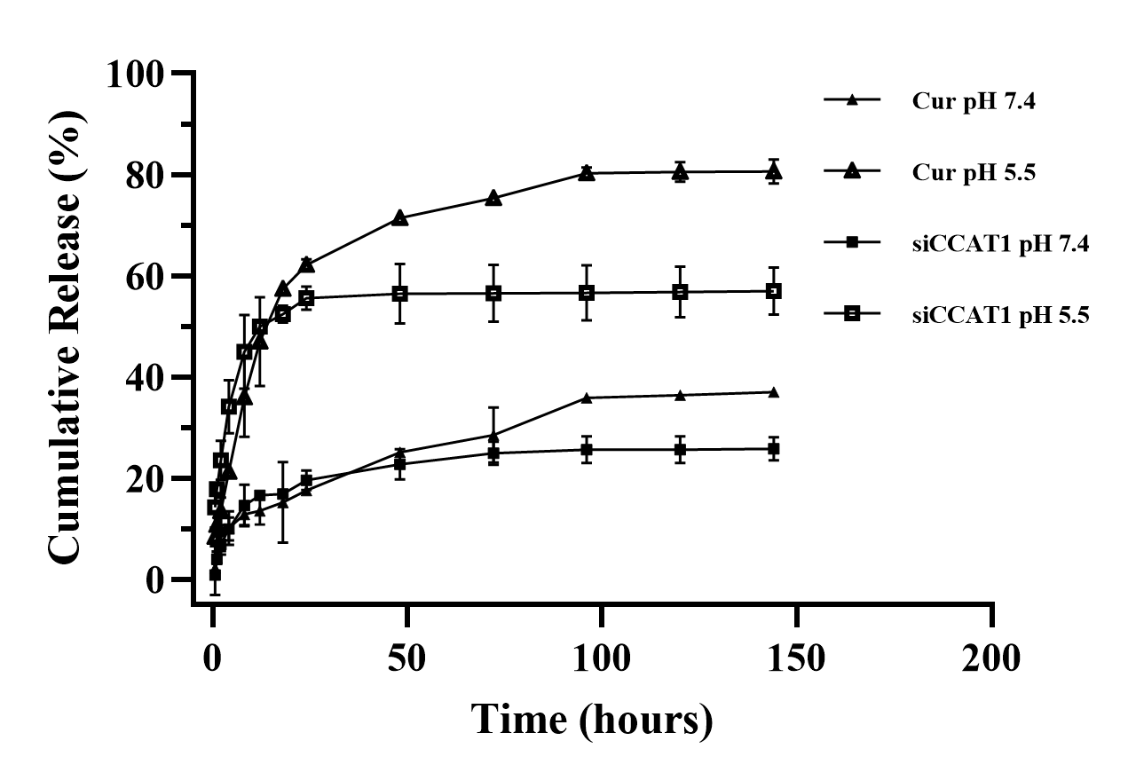


Fig. S6. In vitro Cur and siCCAT1 release profiles from CSNP in PBS at pH 5.5 and pH 7.4 at 37 ℃, respectively.


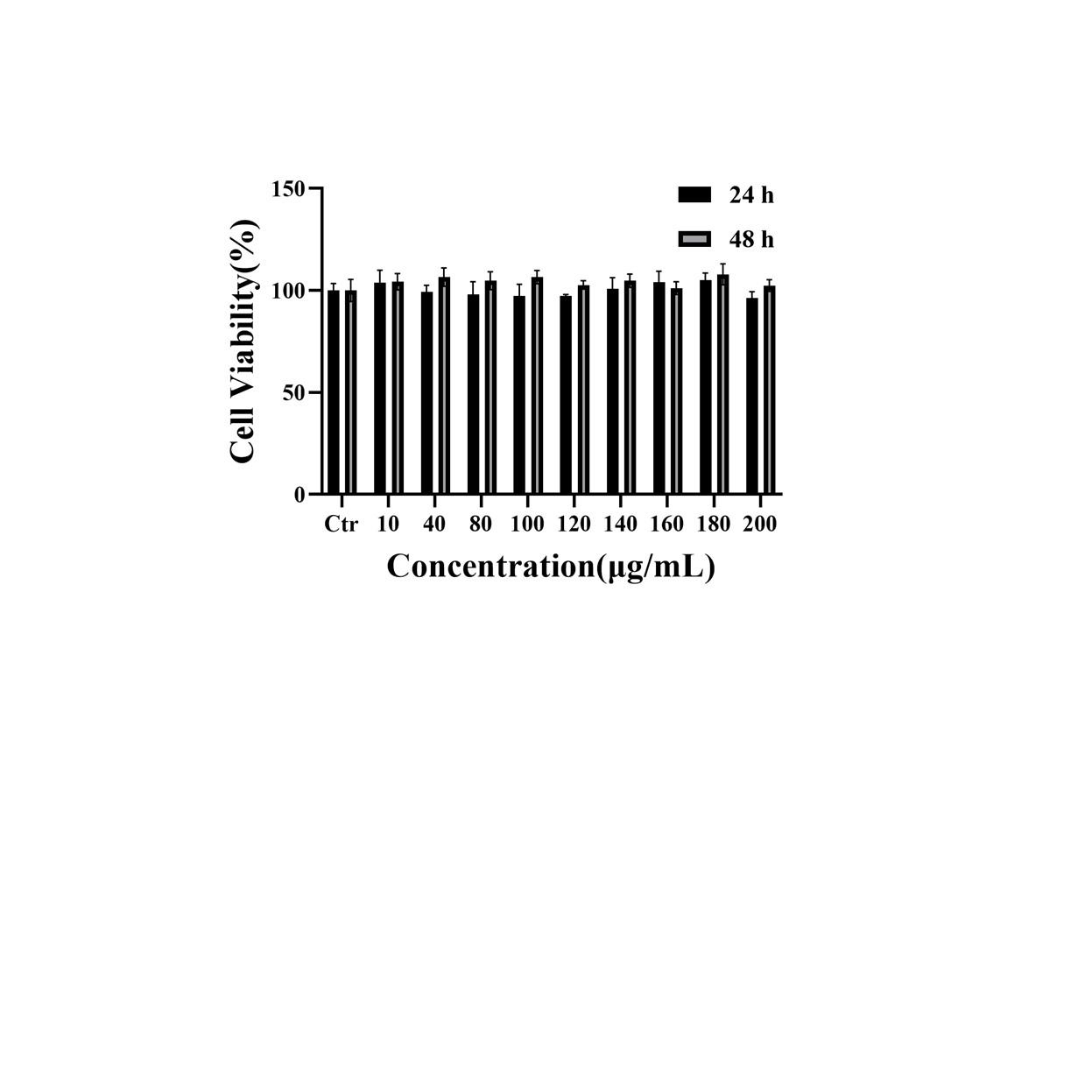


Fig. S7. Cytotoxicity of PEI-PDLLA/DSPE-mPEG blank micelles.


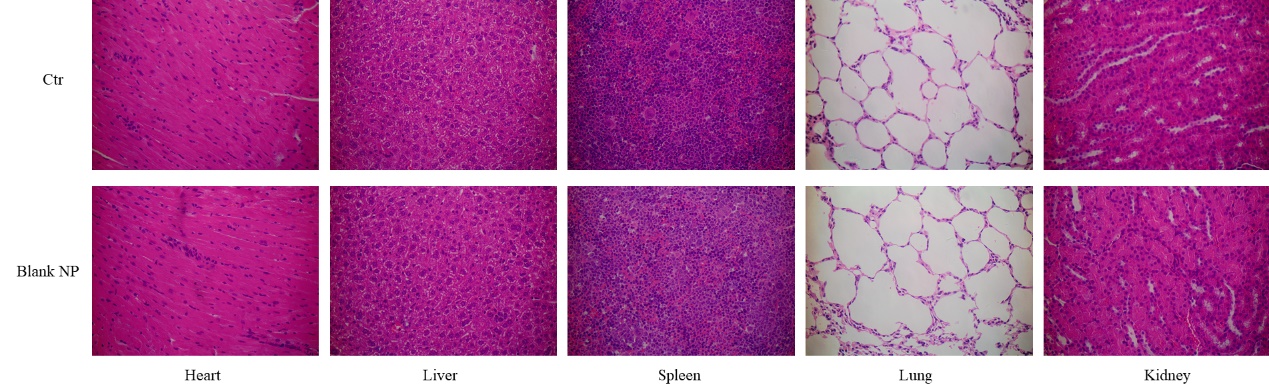


Fig. S8. Histological analysis of main organs with saline and NP treatments, respectively.


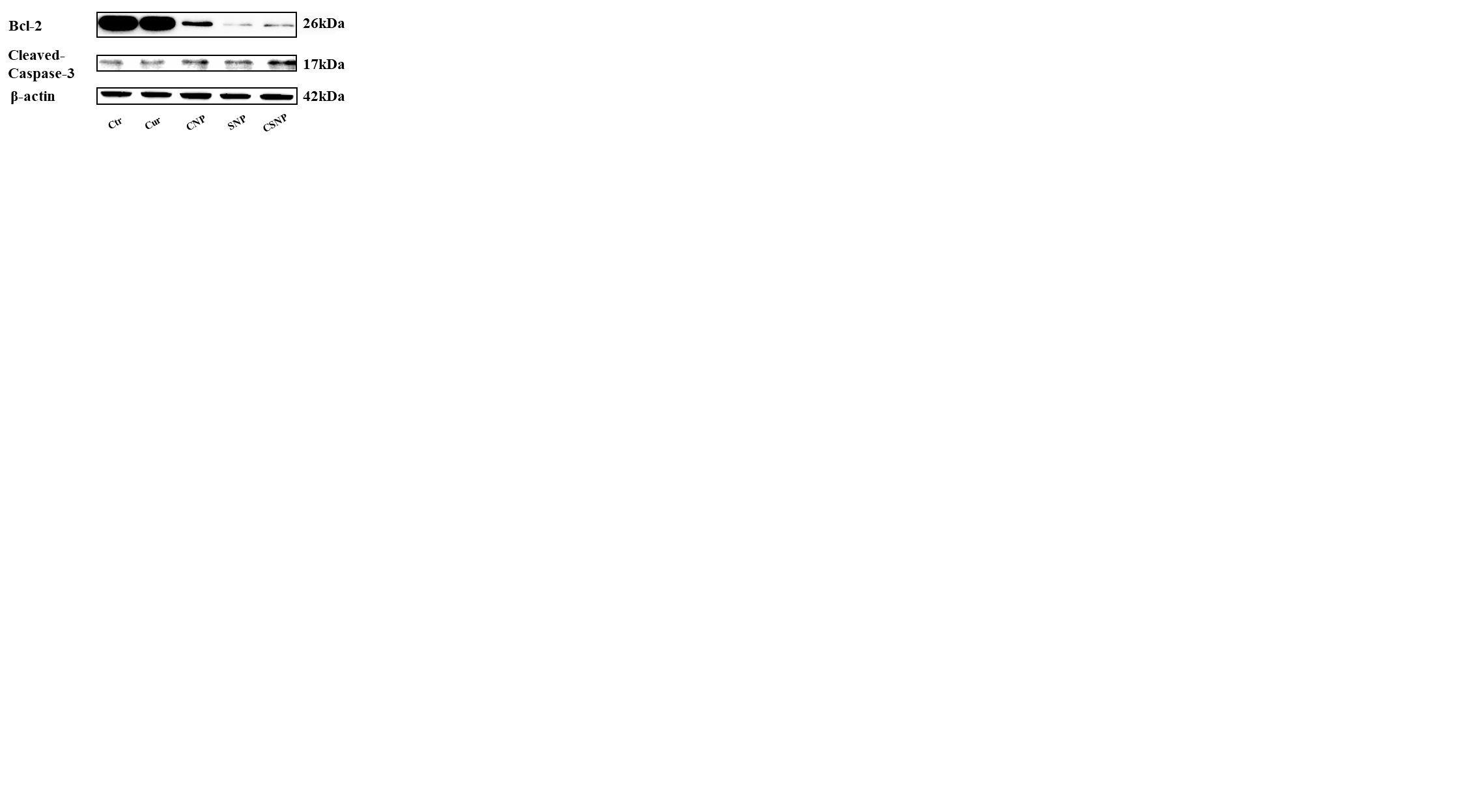


Fig. S9. The protein expression level of Bcl-2 and Caspase-3 of tumor tissues detected by western blot assay.
